# Second Wave of the COVID-19 Pandemic in Delhi, India: High Seroprevalence Not a Deterrent?

**DOI:** 10.7759/cureus.19000

**Published:** 2021-10-24

**Authors:** Nandini Sharma, Pragya Sharma, Saurav Basu, Ritika Bakshi, Ekta Gupta, Reshu Agarwal, Kumar Dushyant, Nutan Mundeja, Zeasaly Marak, Sanjay Singh, Gautam Singh, Ruchir Rustagi

**Affiliations:** 1 Community Medicine, Maulana Azad Medical College, New Delhi, IND; 2 Department of Virology, Institute of Liver and Biliary Sciences, New Delhi, IND; 3 Director General Health Services, Directorate General of Health Services, Government of National Capital Territory, Delhi, New Delhi, IND; 4 Public Health, Directorate General of Health Services, Government of National Capital Territory, Delhi, New Delhi, IND; 5 State Surveillance Unit, Directorate General of Health Services, Government of National Capital Territory, Delhi, New Delhi, IND; 6 State Surveillance Unit, Directorate of Family Welfare, Government of National Capital Territory, Delhi, New Delhi, IND

**Keywords:** antibody titer, pandemic wave, delhi, serosurvey, covid-19

## Abstract

Background

We report the findings of a large follow-up, community-based, cross-sectional serosurvey and correlate it with the coronavirus disease (COVID-19) test-positivity rate and the caseload observed between the peaks of the first and the second wave of the COVID-19 pandemic in Delhi, India.

Methodology

Individuals aged five and above were recruited from 274 wards of the state (population approximately 19.6 million) from January 11 to January 22, 2021. A total of 100 participants each were included from all wards for a net sample size of approximately 28,000. A multistage sampling technique was employed to select participants for the household serosurvey. Anti-severe acute respiratory syndrome coronavirus *2* (SARS-CoV-2) immunoglobulin (IgG) antibodies were detected by using the VITROS® (Ortho Clinical Diagnostics, Raritan, NJ, USA) assay (90% sensitivity, 100% specificity).

Results

Antibody positivity was observed in 14,298 (50.76%) of 28,169 samples. The age, sex, and district population-weighted seroprevalence of the SARS-CoV-2 IgG was 50.52% (95% confidence interval [CI] = 49.94-51.10), and after adjustment for assay characteristics, it was 56.13% (95% CI = 55.49-56.77). On adjusted analysis, participants aged ≥50 years, of female gender, housewives, having ever lived in containment zones, urban slum dwellers, and diabetes or hypertensive patients had significantly higher odds of SARS-CoV-2 antibody positivity.

The peak infection rate and the test-positivity rate since October 2020 were initially observed in mid-November 2020, with a subsequent steep declining trend, followed by a period of persistently low case burden lasting until the first week of March 2021. This was followed by a steady increase followed by an exponential surge in infections from April 2021 onward culminating in the second wave of the pandemic.

Conclusions

The presence of infection-induced immunity from SARS-CoV-2 even in more than one in two people can be ineffective in protecting the population. Despite such high seroprevalence, population susceptibility to COVID-19 can be accentuated by variants of concern having the ability for rapid transmission and depletion of antibody levels with the threat of recurrent infections, signifying the need for mass vaccination.

## Introduction

Monitoring the trends of severe acute respiratory syndrome coronavirus 2 (SARS-CoV-2) constitutes an essential element of the public health response for combating the coronavirus disease 2019 (COVID-19) pandemic [1]. It has been well-established that the number of SARS-CoV-2 infections is several times higher compared to the reported COVID-19 cases because a majority of the infected individuals have an asymptomatic or mild clinical spectrum [2,3]. These subclinical cases may remain untested depending upon the extent of the preparedness of the health system and the performance of contact tracing operations. There is also evidence to suggest that testing strategies based on real-time polymerase chain reaction (PCR) and antigen tests may fail to detect a substantial burden of infections [4,5].

During the COVID-19 pandemic, serosurveys have enabled understanding of the increasing spread of the infection, particularly based on housing settlements because previous serosurveys indicated highly dense, poor urban agglomerates to have a significantly higher burden of SARS-CoV-2 infection compared to low-density, planned settlements [6,7].

In Delhi, the capital city of India, three previous population serosurveys observed the presence of immunoglobulin G (IgG) antibodies to SARS-CoV-2 in 28.39% (August 2020), 24.08% (September 2020), and 24.71% (October 2020) of the population. Moreover, the possibility of waning SARS-CoV-2 IgG seropositivity was suggested on observing the trends of these serosurveys, although contrary evidence was also reported by other studies [8]. A subsequent round of SARS-CoV-2 serosurvey was planned to identify the change in seroprevalence estimates, the variation in the risk factor profile for infection, and the indirect evidence toward recognizing the durability of antibody response.

In this study, we report the findings of a large follow-up, community-based, cross-sectional serosurvey and correlate it with the COVID-19 test-positivity rate and the caseload observed between the peaks of the first and the second wave of the COVID-19 pandemic in Delhi, India.

This article was previously posted to the medRxiv preprint server on September 09, 2021.

## Materials and methods

Study design, participants, and settings

This was a cross-sectional, seroepidemiological study among individuals aged five and above who were recruited from 274 wards in the state of Delhi (population approximately 19.6 million) from January 11 to January 22, 2021.

A total of 100 participants each were enrolled from all wards except the Delhi Cantonment and the New Delhi wards due to their disproportionately larger size. The sample size of approximately 28,000 was estimated at 99% confidence level, 1% absolute precision, 25% expected prevalence from the prior serosurvey [8], design effect of 2, and considering a non-response of 15%. The estimated sample size was also adequately powered to elicit seroprevalence comparisons at the district level.

The housing settlement types in the state of Delhi are classified as a planned colony, an urban slum, a resettlement colony, an unauthorized colony, or a rural village [9]. Resettlement colonies were originally slum-dwelling populations that were resettled in other areas of the city characterized by the unplanned landscape, densely populated, and poor sanitation, albeit an improvement from slum areas. Containment zones were designated clusters of houses in the city-state with a higher frequency of COVID-19 cases, as determined by the local administration with associated regulations restricting the movement of its residents beyond the area perimeter until resolution of the case burden.

Within each ward, the proportion of participants selected from each settlement type was stratified according to their tentatively estimated population size. A multistage sampling technique was employed to select participants for the household serosurvey using the following steps: simple random sampling to select the sampling areas within each settlement type; systematic random sampling to select the households within the selected sampling areas; and selection of individual participants from every selected household using the age-order procedure.

Laboratory procedure

Approximately 3-4 mL of venous blood was collected through venepuncture by a trained phlebotomist or lab technician under aseptic precautions. The samples were transported and processed at the Clinical Virology Lab, Institute of Liver and Biliary Sciences, New Delhi. Anti-SARS-CoV-2 IgG antibodies were detected using the VITROS® assay on VITROS® 3600 (Ortho Clinical Diagnostics, Raritan, NJ, USA) based on chemiluminescent technology, as per the kit literature [10].

Statistical analysis

The sociodemographic data of the participants collected during a brief interview was entered in Microsoft Excel 2013 and merged with their antibody test results. The data were analyzed using SPSS Version 25 (IBM Corp., Armonk, NY, USA) and Stata 14 (StataCorp, College Station, TX, USA). The seroprevalence estimates were weighted to match the state demographics by age and sex and reported as proportions with 95% confidence intervals (CIs). We adjusted the weighted seroprevalence for the assay characteristics using the Rogan-Gladen estimator, where true (adjusted) prevalence = weighted prevalence + (specificity - 1)/(specificity + sensitivity - 1) [11].

The data estimates of COVID-19 cumulative case burden, recovery, and test-positivity rates were obtained from official state government sources. A p-value of <0.05 was considered statistically significant.

## Results

A total of 28,169 laboratory samples were successfully processed excluding 733 samples that were damaged during transit, hemolysis, label mismatch, or inadequate blood collection. The non-response rate at the household level was estimated at 18%. Antibody positivity was observed in 14,298 (50.76%) participants. The age, sex, and district population-weighted seroprevalence of SARS-CoV-2 IgG antibody was 50.52% (95% CI = 49.94-51.10), and after adjustment for assay characteristics, the seroprevalence was estimated to be 56.13% (95% CI = 55.49-56.77).

**Figure 1:**
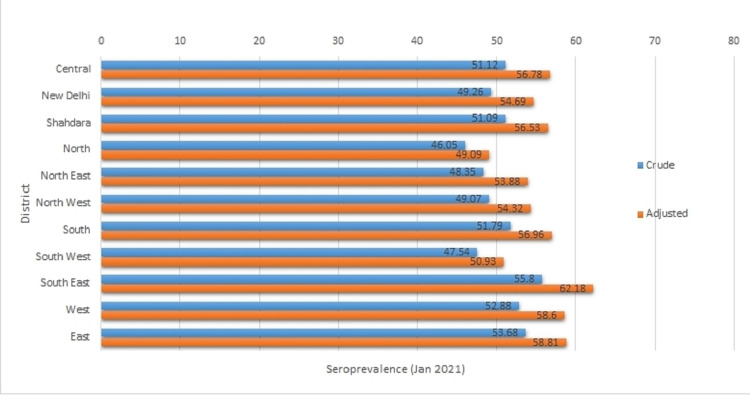
Seroprevalence of SARS-CoV-2 in 11 districts of Delhi, India. Blue: crude; orange: adjusted. SARS-CoV-2: severe acute respiratory syndrome coronavirus 2

The weighted and adjusted seroprevalence (95% CI) in the districts ranged from the lowest in North District 49.09 (46.69-51.51) to the highest in the South-East District 62.18 (60.12-64.21) (Figure 1).

The age and gender-stratified seroprevalence estimates are presented in Table 1. On adjusted analysis, participants aged ≥50 years, of the female gender, housewives, those who had ever lived in containment zones, urban slum dwellers, and diabetes or hypertensive patients had significantly higher odds of SARS-CoV-2 antibody positivity (Table 2). Furthermore, only 72.3% of the participants with a self-reported history of COVID-19 diagnosed by either molecular or antigen methods (n = 1,121) had detectable IgG antibodies to SARS-CoV-2.

**Table 1:** Seroprevalence stratified by district, age, and gender. All calculations were performed on unweighted data. CI: confidence interval

Age	<18 years		18–49 years		≥50 years	
District	Male (%)	Female (%)	Male (%)	Female (%)	Male (%)	Female (%)
Central	54.36	58.60	47.64	49.72	52.26	57.75
New Delhi	54.86	60.13	42.26	49.72	46.55	58.39
Shahdara	52.81	54.31	47.03	49.74	50.79	60.48
North	39.47	44.51	39.53	48.99	50.88	55.21
North-East	50.52	47.06	47.26	50.47	44.50	46.98
North-West	51.93	55.77	44.87	50.20	47.81	49.89
South	49.49	53.81	50.54	54.27	45.45	54.15
South-West	38.55	50.94	46.95	46.13	49.01	51.40
South-East	60.58	60.07	48.58	55.44	56.71	66.08
West	58.85	58.08	47.36	53.03	52.21	60.64
East	53.60	49.26	52.43	51.54	54.87	64.10
Total (%, 95% CI)	51.28 (49.10, 53.45)	54.30 (52.20, 56.39)	46.91 (45.76, 48.06)	50.97 (50.03, 51.91)	50.35 (48.40, 52.29)	56.72 (54.92, 58.51)

**Table 2:** Distribution of sociodemographic and clinical characteristics with IgG SARS-CoV-2 (N = 28,152)*. ^*^17 samples had missing questionnaire data; all analysis were performed on unweighted data. ^+^Column indicating total (N): all percentages are column-wise; column indicating IgG seropositive: all percentages are row-wise COVID-19: coronavirus disease 2019; ILI: influenza-like illness; HTN: hypertension; DM: diabetes mellitus; IgG: immunoglobulin G; SARS-CoV-2: severe acute respiratory syndrome coronavirus 2

Variable	N = 28,152^*^ n, (%)^+^	IgG seropositive^+^	Unadjusted odds	Adjusted odds
Age (years)				
<18	4,337 (15.41)	2,274 (52.43)	1	1
18–49	18,259 (64.88)	8,996 (49.27)	0.88 (0.82, 0.94)	0.89 (0.81, 0.97)
≥50	5,548 (19.71)	2,983 (53.77)	1.06 (0.97, 1.14)	1.03 (0.93, 1.14)
P-value			<0.001	<0.001
Gender				
Male	11,861 (42.13)	5,718 (48.21)	1	1
Female	16,291 (57.87)	8,539 (52.42)	1.18 (1.13, 1.24)	1.16 (1.10, 1.23)
P-value			<0.001	<0.001
Education				
Illiterate	4,958 (17.67)	2,579 (52.03)	1	1
Literate (no formal schooling)	9,774 (34.84)	4,954 (50.69)	0.95 (0.89, 1.01)	0.97 (0.91, 1.05)
Up to 10^th^ class	7,841 (27.95)	3,928 (50.10)	0.93 (0.86, 0.99)	0.96 (0.89, 1.04)
Graduate and above	5,481 (19.54)	2,744 (50.06)	0.92 (0.86, 1.00)	0.95 (0.87, 1.04)
P-value			0.140	0.6915
Occupation				
Self-employed	3,578 (12.75)	1,799 (50.28)	1	1
Salaried	6,192 (22.07)	3,002 (48.48)	0.93 (0.86, 1.01)	0.94 (0.87, 1.03)
Daily wager	2,045 (7.29)	934 (45.65)	0.83 (0.74, 0.93)	0.86 (0.77, 0.96)
Healthcare worker	1,449 (5.17)	750 (51.42)	1.06 (0.94, 1.20)	1.01 (0.89, 1.15)
Housewife	9,325 (33.24)	4,888 (52.42)	1.09 (1.01, 1.18)	1.02 (0.93, 1.11)
Student	5,464 (19.48)	2,833 (51.85)	1.06 (0.98, 1.16)	1.03 (0.93, 1.14)
P-value			<0.001	0.0136
Overcrowding				
Present (≥3 people per room)	9,435 (33.65)	4,799 (50.86)	1.01 (0.96, 1.06)	1.02 (0.97, 1.08)
Absent	18,604 (66.35)	9,402 (50.54)	1	1
P-value			0.606	0.401
Total monthly family income (in Indian Rupees)				
<20,000	15,798 (56.47)	8,006 (50.68)	1	1
20,000–50,000	7,455 (26.65)	3,829 (51.36)	1.03 (0.97, 1.09)	1.03 (0.97, 1.09)
>50,000	4,721 (16.88)	2,331 (49.38)	0.95 (0.89, 1.01)	0.94 (0.87, 1.01)
P-value			0.101	0.0474
Toilet facility				
Individual	7,822 (27.78)	3,934 (50.29)	1	1
Shared	19,269 (68.45)	9,816 (50.94)	1.03 (0.97, 1.08)	1.07 (1.01, 1.13)
Community	1,060 (3.77)	507 (47.79)	0.90 (0.80, 1.03)	0.92 (0.80, 1.05)
P-value			0.103	0.0088
Settlement type				
Planned	9,655 (34.47)	5,010 (51.89)	1	1
Resettlement	1,701 (6.07)	851 (50.03)	0.93 (0.84, 1.03)	0.93 (0.84, 1.04)
Urban slum	8,844 (31.58)	4,639 (52.45)	1.02 (0.97, 1.08)	1.05 (0.98, 1.11)
Unauthorized	3,363 (12.00)	1,662 (49.43)	0.91 (0.84, 0.98)	0.91 (0.84, 0.99)
Village	4,445 (15.87)	2,019 (45.42)	0.77 (0.72, 0.83)	0.78 (0.73, 0.84)
P-value			<0.001	<0.001
History of living in containment zone				
Yes	1,383 (4.93)	797 (57.63)	1.35 (1.21, 1.50)	1.13 (1.00, 1.26)
No	26,669 (95.07)	13,408 (50.28)	1	1
P-value			<0.001	0.045
History of COVID-19 positivity				
Yes	1,121 (4.01)	817 (72.88)	2.72 (2.38, 3.11)	2.95 (2.55, 3.41)
No	26,850 (95.99)	13,345 (49.70)	1	1
P-value			<0.001	<0.001
ILI symptoms				
Yes	599 (2.13)	315 (52.59)	1.08 (0.92, 1.27)	0.80 (0.67, 0.95)
No	27,553 (97.87)	13,942 (50.60)	1	1
P-value			0.336	0.010
HTN				
Yes	805 (2.86)	444 (55.16)	1.21 (1.05, 1.39)	0.91(0.77, 1.06)
No	27,347 (97.14)	13,813 (50.51)	1	1
P-value			0.009	0.229
DM				
Yes	785 (2.79)	472 (60.13)	1.49 (1.29, 1.72)	1.32 (1.12, 1.55)
No	27,367 (97.21)	13,785 (50.37)	1	1
P-value			<0.001	0.001

**Figure 2:**
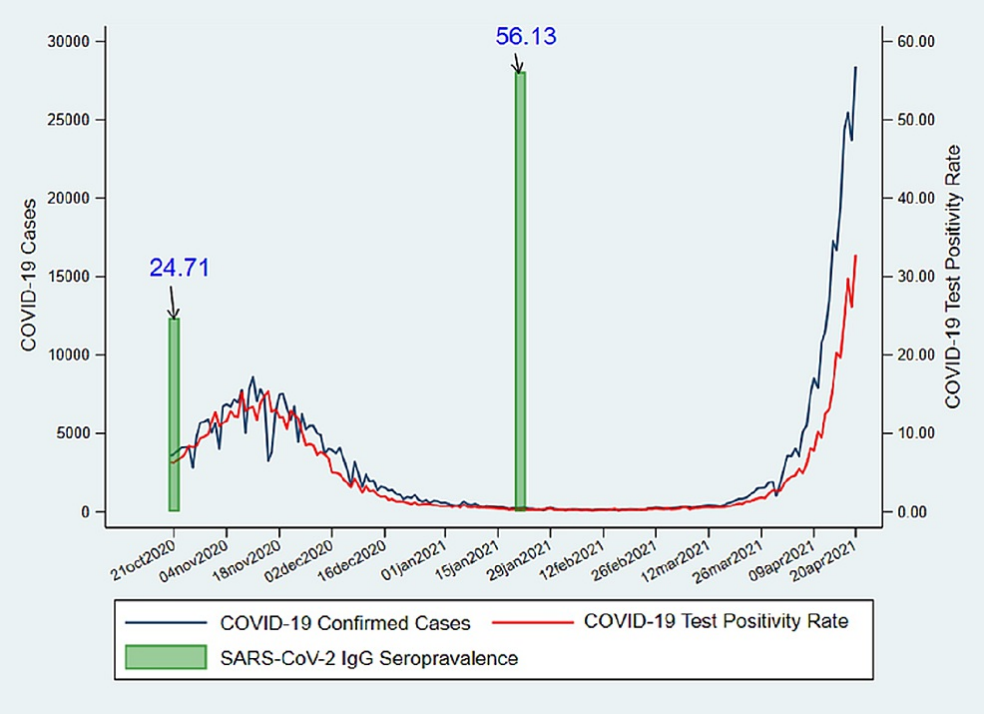
Epidemic curve and seroprevalence of SARS-CoV-2 in Delhi (October 2020 to April 2021). SARS-CoV-2: severe acute respiratory syndrome coronavirus 2; IgG: immunoglobulin G; COVID-19: coronavirus disease 2019

The infection fatality ratio per 1,000 cases ranged from 0.98 (0.96, 1.00) to 0.98 (0.97, 1.01), and the infection to case ratio per 1,000 cases ranged from 56.98 (56.84, 57.12) to 57.31 (57.17, 57.45). The peak infection rate and the test-positivity rate since October 2020 were observed in mid-November 2020, with a subsequent steep declining trend, followed by a period of persistently low case burden lasting until the first week of March 2021. This was followed by a steady increase and subsequently by an exponential surge in infections from April 2021 onwards, indicating the second wave of the pandemic (Figure 2).

## Discussion

In this serosurvey, a majority of the participants had detectable antibodies to SARS-CoV-2 from past infections with nearly uniform district-level trends. In comparison to the three months preceding the survey [8], the antibody seroprevalence in January showed a more than two times increase, coinciding with a rapid decline in the test-positivity rate and the daily new incident cases, suggestive of a high population-level immunity. However, the high seroprevalence through natural infection was insufficient to achieve herd immunity and avert the next wave of the pandemic in Delhi since November 2020, with nearly 0.737 million cases including 11,075 deaths recorded from April to May 2021 [12]. The possibility of a resurgence of cases despite high seroprevalence has been reported from Manaus in Brazil [13]. Furthermore, current research shows that COVID-19 variants of concern B.1.617.2 (Delta) and B.1.1.7 (Alpha), which have more than two times higher transmissibility and have evolved immune escape mechanisms to potentially bypass antibody response induced from natural infection or vaccines, were chiefly responsible for the surge of cases throughout India including Delhi [14,15].

In this study, nearly one in four participants with a history of COVID-19 disease were seronegative, a finding consistent with previous serosurveys where antibodies to COVID-19 were lacking in a substantial proportion of the participants [8]. These findings could be attributed to the growing evidence, suggestive of the waning of IgG antibodies, especially during asymptomatic and mild illness, although the risk of COVID-19 reinfection in previously infected patients remains low [16,17].

The present study has some important implications for public health management of the COVID-19 pandemic, particularly in densely populated lower-middle-income countries. First, the presence of infection-induced immunity even in more than one in two people can be ineffective in protecting the population from large-scale COVID-19-related morbidity and mortality. Second, nearly one in two participants in the less than 18 age group was seropositive, which may have implications toward the opening of educational institutions, reflecting no additional risk in children from exposure to the infection. Third, tracking the emergence of potential variants of concern through robust genomic surveillance and associated contact tracing is necessary [18]. Finally, rapid COVID-19 vaccination with the highest possible coverage remains the most feasible means for mitigating the COVID-19 pandemic. Nevertheless, emerging evidence suggests natural infections confer a significantly more durable and protective immune response against the Delta strain compared to vaccination [16]. Furthermore, individuals with hybrid immunity due to a history of infection prior to COVID-19 vaccination after an interval are known to generate a comprehensive immune response [19]. This signifies the need for universal COVID-19 vaccination, irrespective of the history of SARS-CoV-2 infection, serial assessment of antibody response to identify the potential waning of antibodies, and the necessity of booster doses if required.

This study has certain study limitations. First, nearly one in five eligible individuals from the visited households refused participation in the study. Second, considering the evidence indicative of waning of IgG antibodies to N protein, the estimated seroprevalence levels are likely to be an underestimation [20]. Finally, this serosurvey was conducted prior to the launch of the COVID-19 vaccination program in India, and, consequently, could not assess post-vaccination seroprevalence in the population.

## Conclusions

A huge surge of COVID-19 cases culminating in a massive second wave occurred in the city of Delhi, despite a majority of the population having evidence of past SARS-CoV-2 infection. Therefore, the presence of infection-induced immunity from SARS-CoV-2 even in more than one in two people can be ineffective in protecting the population. Factors that contributed to the subsequent pandemic wave were possibly the presence of variants of concern having high transmissibility. However, the potential susceptibility to reinfection and the development of symptomatic disease because of the waning of SARS-CoV-2 antibodies, especially in asymptomatic individuals, also warrant further exploration in the context of the overall public health impact. Finally, serosurveys at the district level (subnational and subregional level) may be more appropriate in educating public health policy compared to national and state-level estimates, considering the significant interdistrict variation in the observed seroprevalence.
